# Molecular Basis and Clinical Application of Growth-Factor-Independent In Vitro Myeloid Colony Formation in Chronic Myelomonocytic Leukemia

**DOI:** 10.3390/ijms21176057

**Published:** 2020-08-22

**Authors:** Klaus Geissler, Eva Jäger, Agnes Barna, Michael Gurbisz, Temeida Graf, Elmir Graf, Thomas Nösslinger, Michael Pfeilstöcker, Sigrid Machherndl-Spandl, Reinhard Stauder, Armin Zebisch, Heinz Sill, Leopold Öhler, Rajko Kusec, Gregor Hörmann, Peter Valent

**Affiliations:** 1 Medical School, Sigmund Freud University, 1020 Vienna, Austria; 2 Department of Internal Medicine V with Hematology, Oncology and Palliative Medicine, Hospital Hietzing, 1130 Vienna, Austria; forschung.hietzing@gmail.com (T.G.); hietzing.forschung@gmail.com (E.G.); 3 Department of Laboratory Medicine, Medical University of Vienna, 1090 Vienna, Austria; eva.jaeger@akhwien.at (E.J.); michael.gurbisz@meduniwien.ac.at (M.G.); gregor.hoermann@meduniwien.ac.at (G.H.); 4 Blood Transfusion Service, Blood Transfusion Service for Upper Austria, Austrian Red Cross, 4020 Linz, Austria; agnes.barna@o.roteskreuz.at; 5 Department of Internal Medicine III, Hanusch Hospital, 1140 Vienna, Austria; thomas.noesslinger@wgkk.at (T.N.); michael.pfeilstoecker@wgkk.at (M.P.); 6 Department of Internal Medicine I with Hematology with Stem Cell Transplantation, Hemostaseology and Medical Oncology, Ordensklinikum Linz Barmherzige Schwestern—Elisabethinen, 4020 Linz, Austria; Sigrid.Machherndl-Spandl@elisabethinen.or.at; 7 Internal Medicine V with Hematology and Oncology, Medical University of Innsbruck, 6020 Innsbruck, Austria; reinhard.stauder@i-med.ac.at; 8 Department of Internal Medicine, Division of Hematology, Medical University of Graz, 8036 Graz, Austria; armin.zebisch@medunigraz.at (A.Z.); heinz.sill@medunigraz.at (H.S.); 9 Department of Internal Medicine/Oncology, St. Josef Hospital, 1130 Vienna, Austria; leopold.oehler@sjk-wien.at; 10 School of Medicine, University of Zagreb, University Hospital Dubrava, 10000 Zagreb, Croatia; Rajko.Kusec@irb.hr; 11 Central Institute of Medical and Chemical Laboratory Diagnostics, Medical University of Innsbruck, 6020 Innsbruck, Austria; 12 Ludwig Boltzmann Institute for Hematology and Oncology (LBI HO), Medical University of Vienna, 1090 Vienna, Austria; peter.valent@meduniwien.ac.at; 13 Department of Internal Medicine I, Division of Hematology and Hemostaseology, Medical University of Vienna, 1090 Vienna, Austria

**Keywords:** CMML, in vitro cultures, CFU-GM, NGS, prognosis, AML

## Abstract

We have originally reported that colony-forming units granulocyte/macrophage (CFU-GM) formation is an in vitro feature of chronic myelomonocytic leukemia (CMML) and a strong predictor for short survival. Elucidation of the molecular basis underlying this in vitro phenomenon could be helpful to define molecular features that predict inferior outcome in patients. We studied the correlation between the mutational landscape and spontaneous colony formation in 164 samples from 125 CMML patients. As compared to wildtype samples, spontaneous in vitro CFU-GM formation was significantly increased in samples containing mutations in *NRAS*, *CBL* and *EZH2* that were confirmed as independent stimulatory factors by multiple regression analysis. Inducible expression of mutated *RAS* but not *JAK2* was able to induce growth factor independence of Ba/F3 cells. Whereas high colony CFU-GM growth was a strong unfavorable parameter for survival (*p* < 0.00001) and time to transformation (*p* = 0.01390), no single mutated gene had the power to significantly predict for both outcome parameters. A composite molecular parameter including *NRAS/CBL/EZH2*, however, was predictive for inferior survival (*p* = 0.00059) as well as for increased risk of transformation (*p* = 0.01429). In conclusion, we show that the composite molecular profile *NRAS/CBL/EZH2* derived from its impact on spontaneous in vitro myeloid colony formation improves the predictive power over single molecular parameters in patients with CMML.

## 1. Introduction

Growth-factor-independent growth has been long considered as a hallmark of advanced malignancy [1]. Primary tumor cells from hematopoietic malignancies and solid tumors have been shown to grow in vitro without the addition of exogenous growth factors [2,3]. More importantly, the clinical relevance of these findings was demonstrated by the fact that spontaneous growth of tumor cells had a profound adverse impact on the survival of patients.

We have originally shown that extensive formation of colony-forming units granulocyte/macrophage (CFU-GM) without the addition of exogenous growth factors is an in vitro characteristic of a subgroup of patients with CMML [4]. This observation has been reproduced by others [5,6] and seems to be an in vitro phenomenon, which is typical for CMML since it can be regularly demonstrated in CMML but is not a common finding in other MPNs including CML. High spontaneous CFU-GM growth (≥100/10^5^ MNC) was found in 40% of CMML patients in a small retrospective study including 30 patients [7]. Moreover, CMML patients with high spontaneous CFU-GM growth had a much worse prognosis than patients with low colony growth in this study, indicating a clinical significance of our observation. Recently, we were able to demonstrate a close correlation between increased spontaneous colony formation in CMML patients and the presence of RAS-pathway mutations [8]. Moreover, we have shown in a retrospective analysis of 337 CMML patients a correlation of RAS-pathway mutations and spontaneous myeloid colony growth with progression and transformation [9]. Although these findings suggest that high spontaneous CFU-GM formation may be a functional surrogate of RAS-pathway hyperactivation, a detailed analysis of the molecular basis for growth-factor-independent myeloid colony formation is lacking.

## 2. Results

### 2.1. Spontaneous Myeloid Colony Growth Is a Strong Predictor of Survival and Time to Transformation

**Figure 1:**
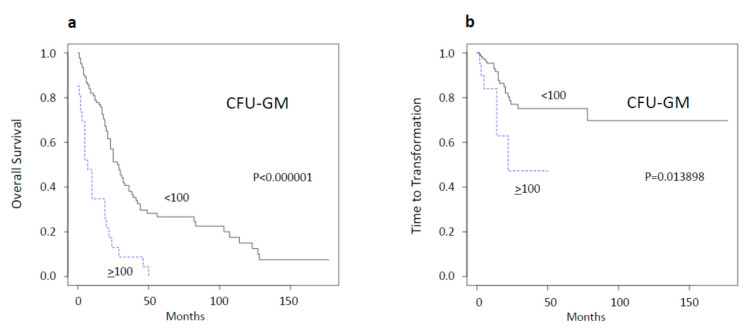
Overall survival (**a**) and time to acute myeloid leukemia transformation (**b**) in chronic myelomonocytic leukemia (CMML) patients stratified by the presence or absence of high (≥100/10^5^ mononuclear cells (MNC)) spontaneous in vitro myeloid colony formation.

In 2004, we reported in a small retrospective study of 30 CMML patients that high spontaneous CFU-GM growth (≥100/10^5^MNC) could be found in a subset of these patients who had a much worse prognosis than patients with low colony growth [7]. Since this time, we have continued to determine spontaneous colony formation in CMML patients, resulting in a current total of 159 patients whose characteristics are shown in Table S1. We now can confirm in this much larger cohort that spontaneous myeloid colony growth is a strong predictor of short survival (Figure 1a). The median survival of patients with high spontaneous myeloid colony growth was 7 months, as compared to 29 months in patients with low growth (<100/10^5^ MNC). High CFU-GM formation did not only predict short survival but also a decreased time to transformation (Figure 1b). By using three patient categories with CFU-GM numbers <20, 20–99, and ≥100 per 10^5^ MNC, respectively, we are also able to discriminate three predictive groups regarding OS and time to transformation, respectively (Figure S1).

The comparison of the predictive power of high spontaneous CFU-GM formation with that of other single established prognostic factors, including white blood cell (WBC) count, hemoglobin (Hb) level, platelet (PLT) count, and the presence of blast cells in peripheral blood (PB), indicates that growth-factor-independent myeloid colony formation had the highest significance regarding overall survival (Table 1). The respective Kaplan Meier plots are shown in the supplementary information (Figure S2).

In a multivariate Cox regression analysis of overall survival, high spontaneous CFU-GM formation remained an independent prognostic factor (Table 2).

### 2.2. Impact of Molecular Aberrations on Spontaneous Myeloid Colony Growth

By having a powerful prognostic in vitro feature available it was of interest to elucidate the molecular basis of this in vitro phenomenon. Therefore, we studied the correlation between the mutational landscape and spontaneous colony formation in 164 samples from 125 CMML patients in whom results from in vitro cultures as well as from NGS analyses were available. In the first step, the Mann-Whitney-U-test was used to compare the spontaneous CFU-GM growth in patients with and without specific molecular aberrations. Table 3 shows the results of these comparisons. As compared to wildtype samples, we found a significant increase in spontaneous in vitro CFU-GM formation in samples containing mutations in *NRAS, CBL* and *EZH2,* and significantly decreased colony growth in *JAK2, TET2* and *IDH1/2* mutated samples. The distributions of CFU-GM values among patients with molecular aberrations are shown by box-plots in Figure S3. In Table S2, the corresponding leukocyte counts are shown. As one can see, the WBC counts from samples with mutations in *NRAS* and *EZH2* were significantly higher than wildtype samples.

In the second step we tried to find out which molecular aberrations had an independent impact on spontaneous myeloid colony formation by using multiple regression analysis. Table 4 shows the results of this analysis. As one can see, we found an independent significant stimulatory effect on spontaneous colony formation by mutations in *NRAS, NF1, CBL* and *EZH2* and an inhibitory effect by mutations in *TET2.*

### 2.3. Impact of RAS G12V and JAK2 V617F on Growth Factor Dependence in Ba/F3 Cells

**Figure 2:**
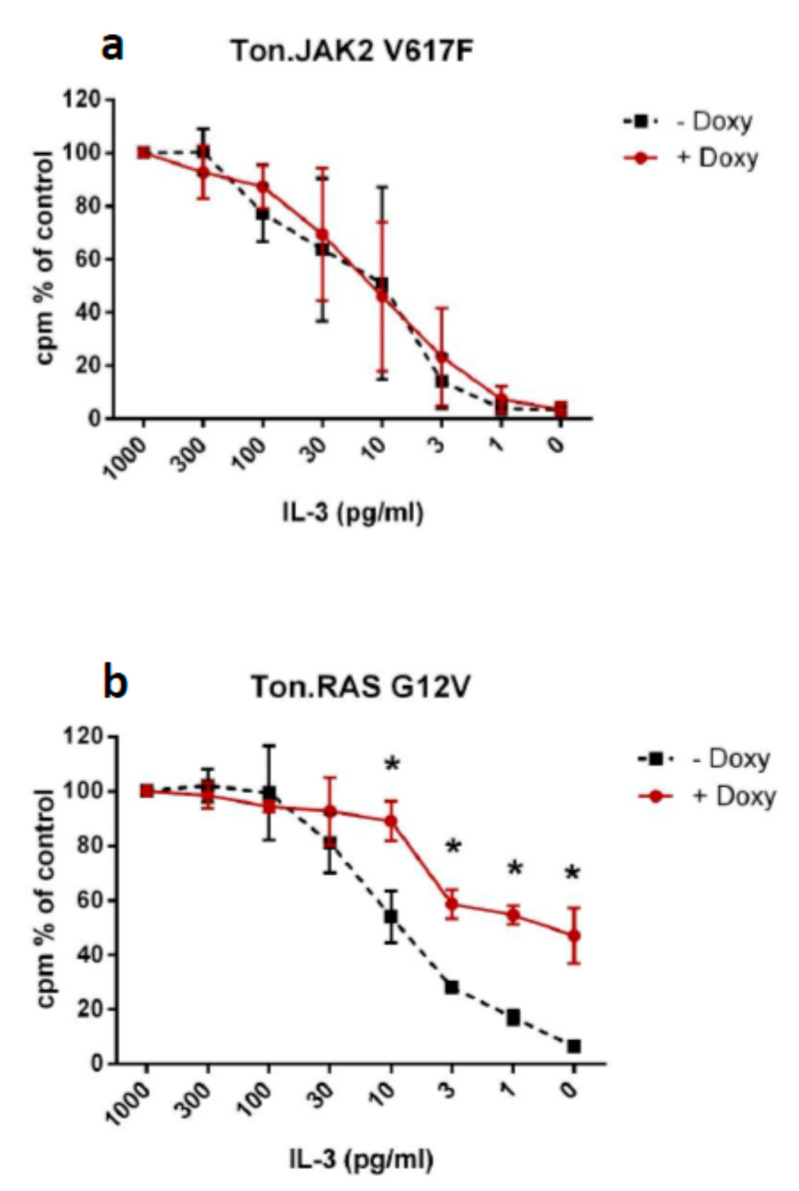
Ba/F3 cells with inducible expression of *JAK2* V617F (**a**) or *RAS* G12V cDNA (**b**) were cultured with or without doxycycline (to induce oncogene expression) in the presence of IL-3. Then, cells were washed and incubated in various concentrations of recombinant murine IL-3 (0–1000 pg/mL) for 48 h. After incubation, proliferation was determined by ^3^H uptake. Results are expressed as percent of control (^3^H-thymidine uptake in the presence of the maximum IL-3 concentration) and represent the mean ± SD of three independent experiments.

In our analysis, aberrations in *NRAS* were by far the most potent stimulator of spontaneous in vitro colony formation. In order to confirm the ability of oncogenic *RAS* to induce growth factor independence in cells, we used an established cell line model and studied the growth factor dependency in Ba/F3 cells with inducible expression of the oncogenes *RAS* G12V or *JAK2* V617F. Figure 1 shows that cells expressing mutated *RAS* were able to grow even with minimal or absent amounts of IL-3 in cell cultures; however, the growth of Ba/F3 cells without oncogene expression and of Ba/F3 expressing mutant *JAK2* was clearly dependent on the presence of IL-3 (Figure 2).

### 2.4. Generation of an in Vitro Myeloid Colony Growth Derived Molecular Pattern and Its Clinical Implication

**Figure 3:**
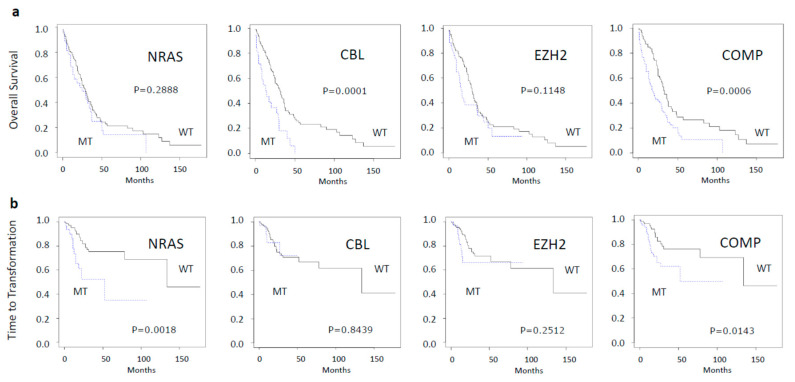
Overall survival (**a**) and time to AML transformation (**b**) in CMML patients stratified by the presence or absence of molecular aberrations. MT—mutated; WT—wildtype; COMP—composite molecular profile including mutations in *NRAS, CBL* or *EZH2.*

Based on our findings, we established a composite molecular pattern including those molecular aberrations that had the greatest impact on spontaneous in vitro CFU-GM growth. Since we wanted to be very restrictive, we included only aberrations that had a significant association with increased colony growth in univariate and multiple regression analysis. When looking at the clinical impact of molecular aberrations, we found that mutations of *NRAS* alone were predictive for time to transformation and mutations of *CBL* were predictive for OS but no single mutated gene had the power to significantly predict for both outcome parameters (Figure 3). Our composite molecular parameter including *NRAS/CBL/EZH2,* however, was predictive for inferior survival (*p* = 0.00059) as well as for increased risk of transformation (*p* = 0.01429).

## 3. Discussion

Since the first description of myeloid colonies in agar from mouse bone marrow by Bradley and Metcalf, semisolid in vitro cultures have made an enormous contribution to our understanding of the regulation of normal and disturbed hematopoiesis [10]. In vitro cultures were the basis for the detection of myeloid growth factors [11]. Moreover, in vitro cultures were very useful in demonstrating aberrant hematopoiesis in many conditions [12]. In normal individuals, the addition of exogenous growth factors is required to stimulate the growth of hematopoietic cell colonies.

We described more than 30 years ago that in a subgroup of patients with CMML, myeloid colonies can grow in vitro without the addition of exogenous growth factors [4]. The basis for this in vitro phenomenon remained poorly understood for a long time. By analyzing a potential correlation between spontaneous colony formation and molecular aberrations in primary cells from patients with CMML, we now provide data indicating that aberrations in the genes *NRAS, CBL* and *EZH2* may be the molecular basis for this in vitro phenomenon.

Molecular aberrations in RASopathy genes have been shown to induce growth factor independence in preclinical mouse models. In the preclinical mouse model, myelomonocytic leukemias can be recapitulated by transplantation of mouse bone marrow cells harboring an oncogenic mutation in the *Nras* locus [13]. Interestingly, alterations of the other RASopathy genes may also lead to a similar phenotype in preclinical mouse models [14,15,16,17]. Mice develop a myeloproliferative disorder with clonal expansion of the granulomonopoiesis in vivo and, importantly, show spontaneous in vitro myeloid colony formation without exogenous growth factors due to aberrant GM-CSF signaling. Here, we show that transfection of oncogenic *RAS* G12V mutation in Ba/F3 causes IL-3 hypersensitivity, confirming the fact that aberrations within the RAS signaling pathway are able to induce growth factor independency.

We cannot exclude the possibility that other mechanisms contribute to the malignant cell growth in CMML. Three common molecular strategies for achieving growth factor autonomy are well established, involving alteration of extracellular growth signals, of transcellular transducers of those signals, or of intracellular circuits that translate those signals into action [1]. Many cancer cells acquire the ability to synthesize growth factors to which they are responsive, creating a positive feedback signaling loop often termed autocrine stimulation [18]. Indeed, we have demonstrated previously that primary CMML cells produce significant amounts of GM-CSF both at the mRNA and protein levels [19]. The fact that Anti-GM-CSF antibodies significantly inhibited the spontaneous growth of myeloid colonies in methylcellulose suggests that an autocrine loop involving GM-CSF may act as an additional mechanism for exaggerated cell growth.

The clinical relevance of spontaneous colony formation is supported by the fact that patients with high spontaneous myeloid colony formation have a dismal prognosis. We reported this observation already in 2004 and can now confirm it with a much higher number of patients [7]. We think that spontaneous colony formation is an excellent parameter to determine the malignant potential of CMML. This parameter is not confounded by death from other causes, as compared to the overall survival, which is commonly used and can be determined at any time point from peripheral blood. Unfortunately, in vitro cultures are labor intensive, are not well standardized, and are not available in many institutions. Therefore, we aimed to use this in vitro phenomenon as a basis to develop a molecular pattern that can be determined more easily by NGS and may reflect this biological phenomenon. This in-vitro-growth-derived composite molecular profile *NRAS/CBL/EZH2* improves the predictive power over single molecular parameters in patients with CMML, and therefore could be useful for managing patients with this malignancy.

## 4. Patients and Methods

### 4.1. Patients

We employed data from 225 CMML patients from the Austrian Biodatabase for Chronic Myelomonocytic Leukemia (ABCMML), which has been shown to be a representative and useful real-life data source for biomedical research [20]. In this database, we retrospectively collected epidemiologic, hematologic, biochemical, clinical, immunophenotypic, cytogenetic, molecular and biologic data of patients with CMML from different centers. Clinical and laboratory routine parameters were obtained from patient records. A detailed central manual retrospective chart review was carried out to ensure data quality before analysis of data from institutions. Data curation included the extraction of discrete data elements from patient records, a check for accuracy and consistency of data, and a verification that baseline data were reflective of CMML that was strictly defined according to WHO criteria [21]. Using 164 MNC samples from 125 patients from the ABCMML, we studied the correlation between the mutational landscape and spontaneous colony formation in patients with CMML. This research has been approved by the ethic committee of the City of Vienna on 10 June 2015 (ethic code: 15-059-VK).

### 4.2. Colony Assay

In one of our centers (Medical University of Vienna), the assessment of hematopoietic colony formation in vitro has been an integral part of the diagnostic work up in patients with suspected myeloid malignancies for many years [12]. Colony-forming unit-granulocyte-macrophage (CFU-GM) growth was assessed in semisolid cultures without growth factors, as previously described in one central laboratory [19]. Mononuclear cells (MNC) were isolated from PB of patients by Ficoll–Hypaque density gradient centrifugation (density 1.077 g/mL, 400× *g* for 40 min). The low-density cells were collected from the interface between density solution and plasma, washed twice, and resuspended in Iscove‘s modified Dulbecco‘s medium (GIBCO, Paisley, Scotland). In unstimulated cultures, PBMNCs were cultured in 0.9% methylcellulose, 30% fetal calf serum (FCS; INLIFE, Wiener Neudorf, Austria), 10% bovine serum albumin (Behring, Marburg, Germany), α-thioglycerol (10^−4^ mol/L) and Iscove‘s modified Dulbecco‘s medium. Cultures were plated in duplicates or triplicates, respectively, at 25–100 × 10^3^ PBMNC/mL. In some cases, the numbers of MNC chosen in our experiments were based on the colony growth in prior cell cultures in the respective patient in order to optimize evaluation of CFU-GM formation. Plates were incubated at 37 °C, 5% CO_2_ and full humidity. After a culture period of 14 days, cultures were examined under an inverted microscope. Aggregates with more than 40 translucent, dispersed cells were counted as CFU-GM. CFU-GM data are expressed as mean values from cultures.

### 4.3. Molecular Studies

Genomic DNA was isolated from mononuclear cell (MNC) fractions of these blood samples according to standard procedures. The mutational status of CMML-related protein coding genes was determined by targeted amplicon sequencing using the MiSeq platform (Illumina, San Diego, CA, USA). Details regarding gene panel, library preparation and data processing have been reported previously [20]. Only variants with strong clinical significance according to the Standards and Guidelines for the Interpretation and Reporting of Sequence Variants in Cancer and VAF ≥ 5% were used for statistical analysis of the impact of molecular aberrations on spontaneous myeloid colony growth [22].

### 4.4. Ba/F3 Cells with Inducible Expression of RAS G12V and JAK2 V617F

In this study, we used the Ba/F3 cell line because this model is a well-established model in many laboratories, including ours, for transfection experiments. The generation of Ba/F3 cells with doxycycline-inducible expression of gene mutations of interest has been described [23]. In brief, Ba/F3 cells expressing the reverse tet-transactivator were cotransfected with pTRE2 vector (Clontech, Palo Alto, CA, USA) containing *HRAS* G12V cDNA and *JAK2* V617F, respectively, and pTK-Hyg (Clontech) by electroporation. Stably transfected cells were selected by growing in hygromycin and cloned by limiting dilution. As assessed by Western blotting, expression of RAS G12V and JAK2 V617F can be induced in Ton.-RAS G12V cells and Ton.JAK2 V617F cells within 12 h by exposure to doxycycline (1 μg/mL).

### 4.5. Measurement of ^3^H-thymidine Uptake

Ton.RAS G12V cells and Ton.JAK2 V617F cells were incubated with or without doxycycline (1 μg/mL) in the presence of recombinant murine IL-3 (1 ng/mL). Then, cells were washed and cultured at different doses of recombinant murine IL-3 in 96-well culture plates (TPP) at 37 °C for 48 h. After incubation of cells, 0.5 μCi ^3^H-thymidine was added (37 °C, 12 h). Cells were then harvested on filter membranes (Packard Bioscience, Meriden, CT, USA) in a Filtermate harvester (Packard Bioscience). Filters were then air-dried, and the bound radioactivity was counted in a β-counter (Top-Count NXT; Packard Bioscience). All experiments were performed in triplicate.

### 4.6. Statistical Analysis

The log-rank test was used to determine whether individual parameters were associated with overall survival (OS) and time to AML transformation. OS was defined as the time from sampling to death (uncensored) or last follow up (censored). Time to AML transformation was defined as the time of sampling to the time of transformation to secondary AML (uncensored) or death/last contact (censored). Dichotomous variables were compared between different groups with the use of the chi-square test. The Mann–Whitney-U-test was used to compare two unmatched groups when continuous variables were not normally distributed. Multiple regression analysis was used to examine how molecular aberrations contribute to growth-factor-independent in vitro myeloid colony formation. Results were considered significant at *p* < 0.05. Statistical analyses were performed with the SPSS v. 19.0.0 (SPSS Inc, Chicago, IL, USA); the reported *p* values were two-sided.

## 5. Conclusions

We show that spontaneous colony formation in CMML functionally covers NRAS and CBL, the most frequent RASopathy gene mutations (>10%), and thus may be considered as a functional parameter for RAS-pathway hyperactivation in these patients. Moreover, the in-vitro-growth-derived composite molecular profile NRAS/CBL/EZH2 improves the predictive power over single molecular parameters in patients with CMML.

## Figures and Tables

**Table 1:** Single prognostic parameters in patients with chronic myelomonocytic leukemia.

Factors	Factors Present Md OS (mo)	Factors Absent Md OS (mo)	Chi-Square Statistics	*p*-Value
CFU-GM ≥ 100/10^5^ MNC	7	29	28.082379	1.162596 × 10^−7^
WBC ≥ 13 G/L	20	29	9.922073	0.001633
Hb < 10 g/dL	17	25	7.288609	0.006939
PLT < 100 G/L	19	29	9.656587	0.001887
PB Blasts present	17	29	18.088790	0.000021
-	Med LFS	Med LFS	-	-
CFU-GM ≥ 100/10^5^ MNC	22	-	6.050997	0.013898
WBC ≥ 13 G/L	-	-	0.520107	0.470796
Hb < 10 g/dL	-	-	0.609919	0.434818
PLT < 100 G/L	-	-	0.320680	0.571199
PB Blasts present	-	-	6.702843	0.009626

**Table 2:** Multivariate Cox regression analysis of overall survival.

Parameter	Hazard Ratio	95% Confidence Interval	*p*-Value
CFU-GM ≥ 100/10^5^ MNC	1.485	1.145–1.1926	0.003
WBC ≥ 13 G/L	1.515	0.946–2.426	0.084
Hb < 10 g/dL	1.348	0.818–2.222	0.241
PLT < 100 G/L	1.602	1.060–2.422	0.025
PB Blasts present	1.413	0.850–2.351	0.183

**Table 3:** Colony-forming units granulocyte/macrophage (CFU-GM) formation per 10^5^ MNC in mutated and wildtype samples of patients with chronic myelomonocytic leukemia.

Genes	Mutated Samples Md CFU-GM (Q1–Q3)	Wildtype Samples Md CFU-GM (Q1–Q3)	*p*-Value Mann-Whitney
*NRAS*	176 (25–264)	7 (0–32)	<00001
*CBL*	35 (17–229)	10 (0–63)	0.0114
*EZH2*	39 (8–389)	10 (1–58)	0.02926
*ASXL1*	31 (2–204)	9 (1–51)	0.05118
*RUNX1*	113 (2–247)	10 (1–52)	0.05876
*SF3B1*	194 (0–285)	11 (1–63)	0.29834
*KRAS*	17 (1–76)	11 (1–109)	0.75656
*SRSF2*	8 (1–136)	12 (1–84)	0.86502
*NF1*	4 (0–216)	11 (1–99)	0.71884
*DNMT3A*	6 (0–139)	13 (1–104)	0.57548
*ZRSR2*	15 (0–40)	12 (1–109)	0.47152
*SETBP1*	6 (0–173)	13 (1–98)	0.42952
*TP53*	4 (0–61)	12 (1–109)	0.33706
*U2AF1*	9 (0–33)	13 (1–109)	0.29834
*PTPN11*	0 (0–180)	13 (1–102)	0.11876
*IDH*	0 (0–12)	13 (1–109)	0.03156
*TET2*	9 (0–52)	37 (4–195)	0.02382
*JAK2*	3 (0–11)	16 (1–116)	0.00854

**Table 4:** Multiple regression analysis on the impact of molecular aberrations on spontaneous in vitro CFU-GM growth.

Genes	Regression Coefficient B	S.D.	2-Tail *p*-Value
*NRAS*	4.51	1.06	4.57 × 10^−5^
*NF1*	4.26	1.54	0.0067
*CBL*	2.27	0.95	0.0188
*EZH2*	2.05	0.83	0.0154
*SF3B1*	4.38	3.45	0.2073
*KRAS*	2.45	1.99	0.2212
*RUNX1*	1.83	1.61	0.2580
*ASXL1*	0.87	1.16	0.4546
*U2AF1*	0.49	1.51	0.7441
*PTPN11*	0.13	2.57	0.9584
*SETBP1*	0.05	0.73	0.9408
*SRSF2*	−0.15	0.95	0.8748
*JAK2*	−0.85	0.89	0.3406
*IDH1/2*	−1.09	2.39	0.6495
*ZRSR2*	−1.03	0.90	0.2541
*TP53*	−1.82	2.95	0.5387
*DNMT3A*	−1.97	1.50	0.1937
*TET2*	−1.29	0.64	0.0484

## Supplementary Materials

Supplementary materials can be found at https://www.mdpi.com/1422-0067/21/17/6057/s1. Table S1: Characteristics of 159 patients with chronic myelomonocytic leukemia; Table S2: White blood cell counts (WBC) in mutated and wildtype samples of patients with chronic myelomonocytic leukemia; Figure S1: Overall survival and time to AML transformation in CMML patients stratified by the number spontaneously formed myeloid colonies (CFU-GM); Figure S2: Overall survival and time to AML transformation in CMML patients stratified by the presence or absence of established prognostic parameters; Figure S3: growth-factor-independent myeloid colony (CFU-GM) formation per 10^5^ MNC in samples of CMML patients with molecular aberrations with VAF < 5%, VAF 5–19% and VAF ≥ 20%.
