# Supplementary material for: Molecular Basis and Clinical Application of Growth-Factor-Independent In Vitro Myeloid Colony Formation in Chronic Myelomonocytic Leukemia
Source: Int J Mol Sci. 2020 Aug 22;21(17):6057. doi: 10.3390/ijms21176057 (PMC7504428; doi:10.3390/ijms21176057)
Supplement: Supplementary file 1 [file ijms-21-06057-s001.pdf]

International Journal of Molecular Sciences

MDPI

Supplementary Materials

Table S1. Characteristics of 159 patients with chronic myelomonocytic leukemia.

Parameters	Values
Age years, median (range)	73 (36 – 92)
Male/Females %	99/60 (62%)
WBC G/L; median (range)	14.1 (2.7 – 156)
WBC ≥13 G/L	84 (53%)
WBC <13 G/L	75 (47%)
Hb g/dl; median (range)	11.2 (4.3 – 14.8)
Hb <10g/dL	42 (26%)
Hb ≥10 g/dL	117 (74%)
PLT G/L; median (range)	116 (5 – 709)
PLT <100 G/L	70 (44%)
PLT ≥100 G/L	89 (56%)
PB blasts %; median (range)	0 (0 – 17)
PB blasts 5-19%	9 (6%)
PB blasts <5%	150 (94%)

Table S2. White blood cell counts (WBC) in mutated and wildtype samples of patients with chronic myelomonocytic leukemia.

Genes	Mutated Samples Md WBC (Q1-Q3)	Wildtype Samples Md WBC (Q1-Q3)	p-Value Mann-Whitney
NRAS	36.9 (16.5-61.4)	13.0 (6.2-26.7)	<0.00001
CBL	17.6 (10.5-43.9)	14.6 (6.9-33.2)	0.15854
EZH2	31.0 (15.1-47.2)	14.2 (6.9-33.2)	0.02382
ASXL1	19.1 (9.1-40.4)	14.2 (6.9-32.9)	0.29372
RUNX1	22.1 (7.8-35.9)	15.8 (7.1-35.9)	0.75656
SF3B1	34.6 (15.9-50.0)	15.0 (7.1-34.8)	0.20054
KRAS	14.5 (8.6-26.2)	16.4 (6.9-37.4)	0.99202
SRSF2	17.3 (6.9-33.1)	15.0 (7.4-37.5)	0.71884
NF1	27.9 (8.5-56.7)	15.6 (7.3-33.2)	0.33204
DNMT3A	14.6 /10.5-39.1)	16.4 (6.9-35.9)	0.68916
ZRSR2	9.1 (4.3-40.4)	16.5 (7.6-35.9)	0.21870
SETBP1	16.9 (6.7-41.9)	14.6 (7.6-33.8)	0.65272
TP53	11.9 (7.5-40.6)	16.4 (7.1-35.9)	0.74140
U2AF1	16.6 (6.6-62.0)	15.8 (7.4-35.9)	0.96012
PTPN11	50.0 (8.5-74.0)	15.8 (7.1-33.2)	0.22628
IDH	11.4 (4.4-17.7)	16.5 (7.7-37.6)	0.08544
TET2	16.6 (6.7-33.5)	14.6 (10.0-40.4)	0.54850
JAK2	17.4 (10.3-40.4)	15.4 (6.8-35.9)	0.33706

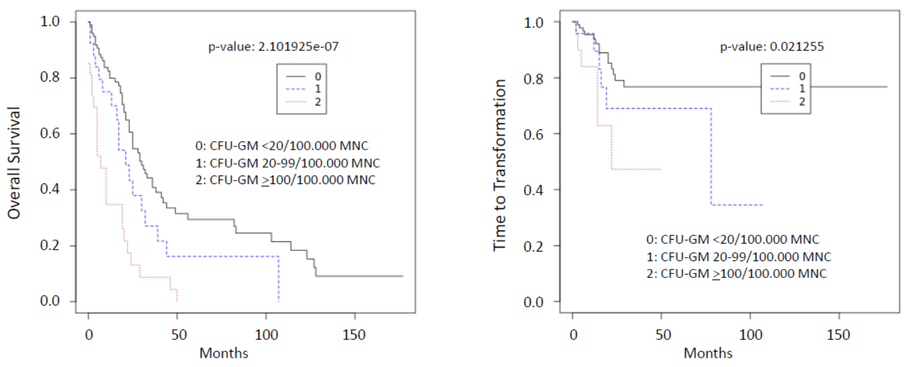

Figure S1. Overall survival and time to AML transformation in CMML patients stratified by the number spontaneously formed myeloid colonies (CFU-GM).

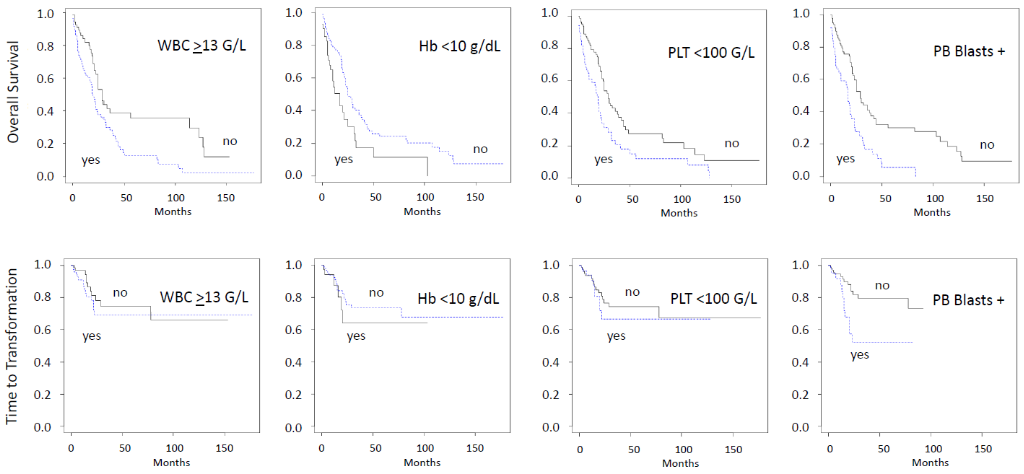

Figure S2. Overall survival and time to AML transformation in CMML patients stratified by the presence or absence of established prognostic parameters.

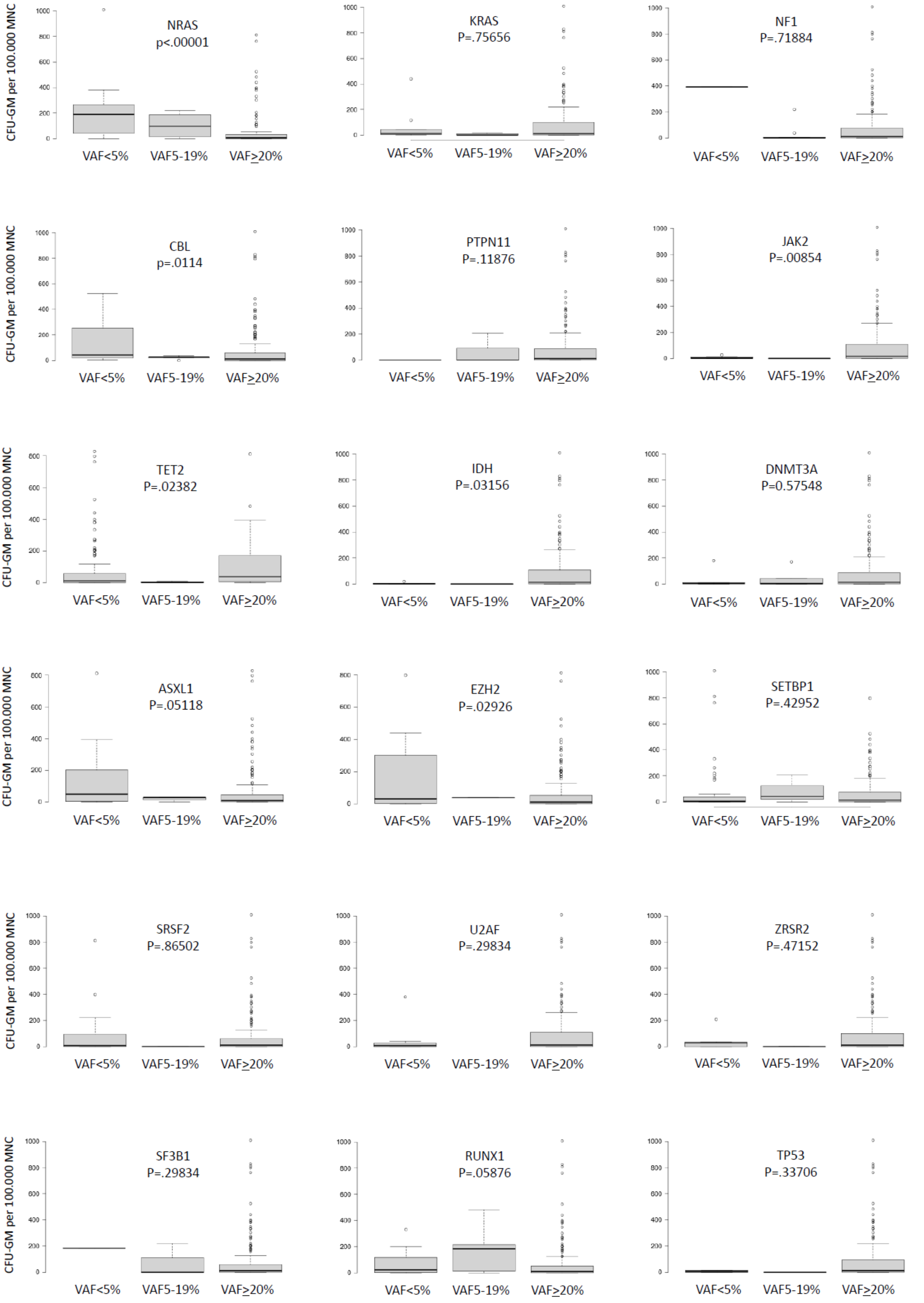

Figure S3. growth factor independent myeloid colony (CFU-GM) formation per 10^5 MNC in samples of CMML patients with molecular aberrations with VAF <5%, VAF 5-19% and VAF ≥20%.
